# Potential challenges to harmonize post-stroke cognitive assessment and its prognostic value: a narrative review

**DOI:** 10.25122/jml-2024-0284

**Published:** 2024-11

**Authors:** Margarita Alexandrova

**Affiliations:** 1 Department of Medical Physics and Biophysics, Medical University-Pleven, Pleven, Bulgaria

**Keywords:** stroke, PSCI, cognitive assessment, prognosis, harmonized criteria, ACE-R, Addenbrooke's Cognitive Examination-Revised;, AUC, area under the curve;, CI, confidence interval;, DSM-5, Diagnostic and Statistical Manual of Mental Disorders, Fifth Edition;, HR, hazard ratio;, ICH, intracerebral hemorrhage;, IST, Isaacs Set Test;, NPV, negative predictive value;, MCI, mild cognitive impairment;, MMSE, Mini-Mental State Examination;, MoCA, Montreal Cognitive Assessment;, mRS, modified Rankin scale;, NIHSS, National Institutes of Health Stroke Scale;, NPV, negative predictive value;, OCS, Oxford Cognitive Screen;, OR, odds ratio;, PPV, positive predictive value;, PSCI, post-stroke cognitive impairment;, SD, standard deviation;, TIA, transient ischemic attack;, VASCOG, Vascular Behavioral and Cognitive Disorders;, VCD,vascular cognitive disorders

## Abstract

With advances in scientific and clinical knowledge, stroke has evolved from a major cause of death to a chronic condition affecting the daily lives of sufferers, their relatives, and society. Post-stroke cognitive impairment (PSCI) is common even among individuals with good neurological recovery. When deciding on interventions aimed to improve the life quality of post-stroke patients, identifying those at high risk of cognitive decline proves crucial. Given the complexity of PSCI assessment, this narrative review discusses the feasibility of developing standardized criteria for selecting cognitive instruments. Potential approaches for establishing harmonized procedures for post-stroke cognitive assessment are presented depending on how the cognitive impairment is defined, the cognitive domains examined, the methods used to generalize cognitive data by components/domains, and their normalization against standardized normative samples. The prognostic value of cognitive assessment to identify patients at high risk of PSCI, functional dependence, and poor survival is also discussed. Implementing harmonized criteria for assessing the cognitive status of stroke patients could reduce the now considerable heterogeneity between studies and serve as a reliable basis for determining the prevalence and predicting the occurrence/aggravation of PSCI.

## INTRODUCTION

The incidence of cerebrovascular accidents and dementia has increased significantly with the aging of the population. Statistically, stroke remains the second leading cause of death worldwide and ranks third as the cause of death and disability combined [1]. The global cost of treating stroke is enormous (0.66% of global GDP) [1]. In addition to motor and sensory disorders, stroke can cause cognitive impairment. Post-stroke cognitive impairment (PSCI) is defined as any cognitive impairment, regardless of its severity and cause, recorded after a clinically confirmed stroke and includes cognitive deficits ranging in severity from mild cognitive impairment to dementia [2-4]. PSCI has been associated with increased mortality, dependency, institutionalization, and low long-term post-stroke survival [5-7]. Furthermore, it is linked to increased health and socioeconomic burden [8]. In contrast, intact cognitive function is a leading factor determining stroke survivors' potential prospects for rehabilitation and recovery.

With improvements in the treatment of acute strokes, a more significant proportion of patients survive. Long-term cognitive impairment, however, is common even after good neurological recovery [9-11]. Thus, stroke has transformed from a major cause of death to a long-term (chronic) condition affecting the everyday lives of patients, their families, and society. Therefore, it is crucial to identify individuals at high risk of cognitive decline after stroke. Early neuropsychological evaluation is essential for assessing cognitive dysfunction and the need for rehabilitation. Adopting harmonized criteria for assessing cognitive status in stroke patients may allow a more accurate determination of the prevalence and prediction of PSCI [12]. Also, it can improve the awareness of relatives about appropriate coping strategies in dealing with the potential social and societal burden generated by cognitive decline, even after minor vascular incidents.

This narrative review discusses issues related to evaluating post-stroke cognitive status, potential approaches to standardize/harmonize cognitive assessment, and its prognostic value.

## MATERIAL AND METHODS

### Search strategy

The search strategy was designed to explore potential opportunities to harmonize PSCI assessment criteria and procedures and to estimate the prognostic value of cognitive assessment across different stroke phases. For this purpose, a search for articles was performed using the MedLine, Scopus, PubMed, and Google Scholar databases. Keywords included 'stroke', 'cognition', 'PSCI', 'dementia', 'vascular', 'cognitive impairment', 'MCI', 'screening', 'neuropsychological assessment', and 'prognosis'.

### Study selection

The inclusion/exclusion criteria chosen were aimed at selecting peer-reviewed publications focused on the assessment of cognitive abilities of patients after stroke and the prognostic value of this assessment. Inclusion criteria were as follows: (1) studies of adult individuals with cognitive impairment (mild or dementia) after confirmed ischemic stroke, hemorrhagic stroke, or transient ischemic attack (TIA), regardless of their severity, time elapsed since stroke, number of strokes experienced, and follow-up period; (2) narrative and systematic reviews, meta-analyses, randomized controlled trials (RCTs), prospective and retrospective cohort studies, validation studies, case-control studies, cross-sectional studies, clinical guidelines, and editorials; (3) materials published in English in peer-reviewed indexed and refereed journals in the last 15 years (2009–2024). Relevant publications missed in the database search but referred to in the already selected articles were also included in the analysis. Priority was given to (1) comparative studies on the diagnostic accuracy and prognostic value of different cognitive assessment tools; (2) manuscripts published in the last 5 years; (3) articles with a higher level of evidence according to the pyramid of evidence-based medicine, in the following order: systematic reviews and meta-analyses, guidelines, RCTs, cohort studies, and case-control studies; (4) articles with larger samples and/or longer follow-up times.

**Figure 1:**
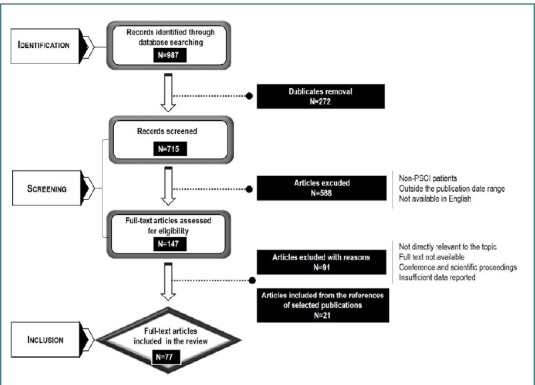
Article selection flowchart

We excluded from the review (1) articles not related to the research aim; (2) articles not available in full text; (3) conference proceedings (abstracts and papers) and scientific proceedings. The selection process of the papers used in the review is presented in Figure 1.

### Included articles

The total number of articles included in the review after applying the inclusion/exclusion criteria was 77. Of these, 16% (*n* = 12/77) were published in the last 5 years, and 18% (*n* = 14/77) were systematic reviews and guidelines at the top of the evidence-based medicine pyramid.

Guidelines for writing narrative reviews were followed in the preparation of the manuscript [13,14].

## POST-STROKE COGNITIVE ASSESSMENT CRITERIA FOR DETERMINING COGNITIVE STATUS

**Figure 2:**
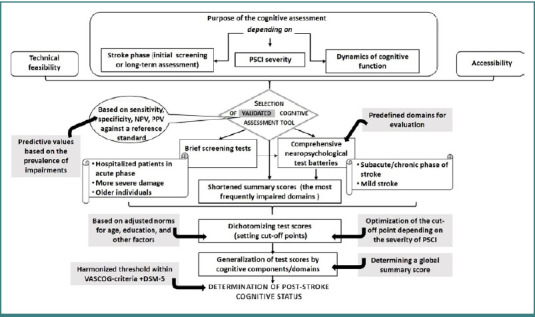
Post-stroke cognitive assessment. The recommended criteria for harmonizing cognitive assessment are represented in gray rectangles. DSM-5, Diagnostic and Statistical Manual of Mental Disorders, Fifth Edition; NPV, negative predictive value; PPV, positive predictive value; PSCI, post-stroke cognitive impairment; VASCOG, Vascular Behavioral and Cognitive Disorders.

It is now increasingly acknowledged that assessment of cognitive performance after stroke should be incorporated into the 'routine' neurological examination in clinical practice and research [9,15,16]. However, the objectivity of cognitive impairment assessment after stroke is still debatable [17–20]. Cognitive assessment is complex and should be based on some standardized criteria, such as diagnostic criteria for defining vascular cognitive impairment, selection of a standardized procedure for assessing post-stroke cognition – the type of cognitive instrument (screening test/battery), a statistical method for determining a cut-off point, criteria for selecting an approach for generalizing cognitive data across components/domains, etc. (Figure 2). Harmonizing criteria for assessing cognitive status is a priority for objectively determining the diagnosis, prevalence, and prognosis of the onset/aggravation of PSCI [7,21].

A set of diagnostic criteria (a VASCOG statement) for defining vascular cognitive disorders (VCD) has been proposed in the literature to stimulate clinically and pathologically validated studies [18]. In addition, harmonizing these criteria with the Diagnostic and Statistical Manual of Mental Disorders, Fifth Edition (DSM-5) criteria has provided a prerequisite for an international consensus on diagnosing VCD [15,18].

### Cognitive assessment tools

Various cognitive instruments have been applied in studies, ranging from brief screening tests to comprehensive neuropsychological test batteries. It is believed that the choice of such a tool should be determined by many factors, such as the purpose of the testing (the degree of cognitive impairment it can diagnose), accessibility, technical feasibility, etc. (Figure 2). For example, if the assessment aims to identify all potential cases of PSCI, a high-sensitivity instrument is needed. Technical feasibility, in turn, is an essential factor, especially in the acute phase of stroke, when the severity of the disease may not allow prolonged neuropsychological testing [15,22].

Since stroke patients may have specific cognitive deficits (e.g., aphasia or neglect) or more global cognitive dysfunction, cognitive testing should include an assessment of cognitive domains. A study of PSCI in the acute phase of stroke showed a high prevalence of impairments in general cognitive ability and the five most commonly assessed domains: complex attention, executive function, learning and memory, language, and perceptual-motor control [23]. There is evidence that even mild post-stroke cognitive impairment is multidomain, so it is necessary to use a combination of different tests to establish the cognitive status [19]. Assessing additional cognitive abilities such as processing speed and abstract reasoning may improve screening since disorders in these two domains significantly predict short- and long-term cognitive and functional impairment [12, 24-26].

A comprehensive neuropsychological assessment that uses reliable and validated instruments to measure multiple cognitive domains is considered the gold standard for evaluating cognitive dysfunction after stroke. An extended neurocognitive battery has been proposed in the literature with a very high sensitivity (91%) [27], which measures language, neglect and praxis, memory and emotional reactions, and screens for several specific cognitive syndromes. However, such an instrument has low specificity (35%) in stroke because patients with more severe impairments perform poorly on longitudinal neuropsychological tests [26]. The National Institute of Neurological Disorders and Stroke - Canadian Stroke Network (NINDS-CSN) neuropsychological battery with predefined cognitive domains for assessment has been developed and applied in research studies [28]. Assessing the same basic cognitive domains is essential to reduce the high heterogeneity between studies [17]. Moreover, Barbay *et al*. [12] suggested optimizing the criteria within the VASCOG statement by evaluating only the most frequently impaired domains and generating the so-called shortened summary score (an averaged score of the data on action speed, executive functions, and language), reporting an increase of testing sensitivity by 9% [12] (Figure 2).

A comprehensive neuropsychological examination takes time and is exhausting for stroke patients. In more severely disabled, older patients or those in the acute phase of stroke, when the cognitive impairment is most apparent, using shorter screening tools is recommended [22,29]. It should be noted that screening tests may omit very mild cognitive decline, which is more likely to benefit from intervention. Hence, a brief screening test can be used for an initial assessment of cognitive status but cannot be a substitute for subsequent multicomponent evaluation (Figure 2).

The Montreal Cognitive Assessment (MoCA), Addenbrooke's Cognitive Examination-Revised (ACE-R), Mini-Mental State Examination (MMSE), Isaacs Set Test (IST), Diagnostic and Statistical Manual of Mental Disorders (DSM), Informant Questionnaire on Cognitive Decline in the Elderly, Rotterdam-CAMCOG (R-CAMCOG), among others, are widely used brief cognitive screening instruments [22,30-36]. They are considered applicable in routine clinical practice and extensive stroke studies. In a meta-analysis, Lees *et al*. [22] reported that four of the above-mentioned cognitive screening tools (ACE-R, MMSE, MoCA, and Rotterdam-CAMCOG) have similar accuracy for detecting PSCI, with none showing marked superiority (Table 1). Moreover, when comparing shorter screening tests, such as MoCA (<22/30), with longer ones, such as ACE-R (<88/100), no significant difference in detecting cognitive impairments was found between their sensitivity (84% and 96%), and specificity (78% and 70%), respectively [22] (Table 1). The MoCA test is, therefore, often preferred when initial screening aims to cover all potential cases of PSCI, as it offers high sensitivity and takes less time than tests with comparable sensitivity.

**Table 1:** Test accuracy of some cognitive screening instruments

Source	Studies (patients), n	Screening tool	Cut-offs	Sensitivity, % (95% CI)	Specificity, % (95% CI)	PPV or PLR^※^ (95% CI)	NPV or NLR^※^ (95% CI)	AUC (95% CI)
Bour A *et al*., 2010 [30]	(194)	MMSE	<27-28/30, ≥1 impaired domain	72	71	0.93		0.79
			<27-28/30, ≥2 impaired domain	80	70	0.86		0.86
			<26-27/30, ≥4 impaired domain	82	75	0.72		0.88
Cumming TB *et al*., 2013 [31]	(60)	MOCA MMSE	<23-24/30 <26-27/30	92(80–97) 82	67(45–83) 76	0.84 0.86	0.82 0.76	0.87(0.78-0.97) 0.84(0.73-0.95)
Dong Y *et al*., 2012 [32]	(239)	MoCA	<21-22/30	88	64	0.45	0.94	0.85(0.79-0.90)
	(239)	MMSE	<25-26/30	88	67	0.47	0.74	0.83(0.77-0.89)
Godefroy O *et al*., 2011 [33]	(95)	MoCA	<22/30	78	90	0.94	0.67	0.89(0.83-0.96)
			<26/30	98	26	0.73	0.89	
		MMSE	<24/30	70	97	0.98	0.61	0.88(0.82-0.95)
			<25/30	73	87	0.92	0.61	
Lees R *et al*., 2014 [22]	6(726)	MOCA	<22/30	84(76–89)	78(69–84)	3.75(2.77–5.08)^※^	0.20(0.15–0.29)^※^	
	4(326)		<26/30	95(89–98)	45(34–57)	1.73(1.43–2.10)^※^	0.10(0.04–0.23)^※^	
	12(1639)	MMSE	<25/30	71(60–80)	85(80–89)	4.73(3.63–6.17)^※^	0.34(0.25–0.47)^※^	
	5(445)		<27/30	88(82–92)	62(50–73)	2.33(1.72–3.17)^※^	0.19(0.13–0.29)^※^	
	2(192)	ACE-R	<88/100	96(90–100)	70(59–80)	3.19(2.24–4.54)^※^	0.06(0.01–0.22)^※^	
	2(421)	R-CAMCOG	<33/49	81(57–93)	92(87–95)	10.18(6.41–16.18)^※^	0.20(0.07–0.52)^※^	
Lees R *et al*., 2014 [34]	(173)	Cog-4 (vs. MoCA-defined impairment)	Cog-4≥1 MoCA<26/30	36(28–45)	96(80–99)	0.98(0.89–1.00)	0.23(0.16–0.32)	
Morris K *et al*., 2012 [35]	(101)	MMSE	<27/30	80(68–89)	20(6–51)	0.84	0.16	0.53
	(101)	ACE-R	<88/100	90	20	0.85	0.28	0.53
Pendlebury *et al*., 2012 [36]	(91)	MOCA	<23/30	49(32–65)	90(79–97)	0.79(0.58–0.93)	0.70(0.58–0.81)	
			<26/30	87(73–96)	63(49–76)	0.64(0.50–0.77)	0.87(0.72–0.96)	
	(91)	MMSE	<26/30	36(21–53)	92(81–98)	0.78(0.52–0.94)	0.66(0.53–0.76)	
	(91)	ACE-R	<88/100	56(38–72)	100(93–100)	1.00(0.83–1.00)	0.75(0.63–0.85)	

ACE-R, Addenbrooke's Cognitive Examination-Revised; AUC, area under the curve; CI, confidence interval; Cog-4, 4 cognitive areas of the National Institutes of Health Stroke Scale; MMSE, Mini-Mental State Examination; MoCA, Montreal Cognitive Assessment; NLR, negative likelihood ratio; NPV, negative predictive value; n – number of studies (patients); PLR, positive likelihood ratio; PPV, positive predictive value.

MMSE is also widely used in epidemiological studies and clinical trials of large samples of patients with PSCI (Table 1). The main difference between the MoCA and MMSE scales is that the latter does not assess executive functions [37].

When comparing the two scales, the MoCA administered in the chronic phase detected more cognitive impairments than the MMSE [38,39] (Table 2). MoCA allowed better discrimination of the cognitive profiles of older adults without stroke, individuals with TIA, or stroke (more than 6 months after the event) [40, 41]. In addition, insufficient sensitivity and specificity of the MMSE scale have been reported [42]. MMSE has lower sensitivity for mild cognitive impairment (MCI) in single-domain disorders [36] (Table 2).

**Table 2:** Comparison between MoCA and MMSE in terms of PSCI detection and/or dynamics

Source	Main results	Time to administer
Delavaran H *et al*. (2017) [39]	MoCA is more suitable than MMSE to register long-term PSCI. MMSE showed PSCI in 46% of patients, whereas MoCA - in 61%. Among the stroke survivors with MoCA<25, 35% had MMSE≥27 (*P*<0.001).	10 years post-stroke
Lees R *et al*. (2014) [22] a meta-analysis	MoCA and MMSE have similar accuracy for detecting dementia/multidomain impairment. MMSE (<27/30): sensitivity 71%, specificity 85% (12 studies); MoCA (<26/30): sensitivity 95%, specificity 45% (4 studies); MoCA (<22/30): sensitivity 84%, specificity 78% (6 studies)	at any time post-stroke
Pendlebury ST *et al*. (2010) [38]	MoCA records more cognitive impairments after TIA/stroke than the MMSE, demonstrating deficits in executive function, attention, and delayed recall.	at a 6-month or 5-year follow-up after TIA/stroke
Pendlebury ST *et al*. (2012) [36]	MoCA has good sensitivity and specificity for MCI, whereas MMSE shows a ceiling effect. Sensitivity (77%) and specificity (83%) for MCI were optimal at MoCA <25. MMSE achieved sensitivity >70% only at a cut-off value <29, mainly because of relative insensitivity to single-domain disorders.	>1 year after TIA/stroke
Sivakumar L *et al*. (2014) [44]	Acute temporary cognitive impairment after TIA/minor stroke is common. Cognitive impairment was registered in 54% with MoCA and 16% with MMSE; P=0.001. MoCA scores improved on days 7, 30, and 90; P<0.0001. The MMSE is not sensitive to these changes.	across 90 days after TIA/stroke
Tan HH *et al*. (2017) [46]	Patients who experienced a decline in MoCA scores from 3–6 months to 1 year were three times more likely to worsen their diagnosis transitional status (OR = 3.21, p = 0.004). No significant relation existed between the MMSE scores decline and having a decline in diagnosis transitional status from 3–6 months to 1 year after TIA/stroke. The MMSE may not be as sensitive as the MoCA in registering cognition changes.	from 3–6 mo to 1 year after TIA/stroke

MCI, mild cognitive impairment; MMSE, Mini-Mental State Examination; MoCA, Montreal Cognitive Assessment; OR, odds ratio; TIA, transient ischemic attack

The Chinese version of MoCA (MoCA-BC) is also considered superior to MMSE in detecting MCI [43]. It should be noted, however, that some meta-analyses suggest that MMSE is a better test for diagnosing multidomain impairments than other screening tools [22] (Table 1).

Another difference between the two scales is their ability to track the dynamics of post-stroke cognitive function [44] (Table 2). In the prospective study of Krishnan *et al*. [45], including patients with mild cognitive impairment, MoCA was able to record temporal cognitive changes over 3.5 years after stroke (M = -1.83, *P* < 0.001, d = 0.64). Tan *et al*. [46] have also concluded that MoCA is a clinically relevant tool for tracking cognitive variations over time (Table 2). On the contrary, MMSE has not been reported as sensitive in following up the dynamics of cognitive status [26,46]. Also, there is published evidence that the sensitivity of MoCA to identify subtle cognitive impairments in patients with cerebrovascular disease is similar to that of the computerized MindStreams neuropsychological test battery [47]. A moderate positive correlation (r = 0.6 *P* < 0.001) was reported between these two rating scales regarding memory, attention, and executive functions. Patients with low MoCA scores (<26/30) also had significantly lower cognitive scores in all MindStreams subcategories (executive function, memory, visual processing, verbal function, and attention) (*P* < 0.001).

The main criticisms of the MoCA and MMSE application in stroke are related to their feasibility, especially in the acute disease stage, when stroke-related impairments like aphasia and hemispatial neglect may influence the scale scores obtained.

Other brief screening tools have been studied to find the most effective method to assess cognitive status. IST is a quick cognitive ability test focusing on executive functions (cognitive set-shifting, generation, and processing speed) and semantic and working memory, which have predictive value for the preclinical detection of dementia. The IST has been reported as a reliable and rapid screening tool for assessing cognitive impairment after delayed-onset stroke [26]. In the review of Lees *et al*. [22], R-CAMCOG is also considered a tool with good clinical applicability (Table 1). However, definite conclusions about the scale cannot be drawn due to the small number of studies using it. Two other brief screening tests are the Cognistat and the Screening Instrument for Neuropsychological Impairment after Stroke, with sensitivities of 82% and 71%, respectively, for recording deficits in any cognitive domain compared with a full neuropsychological assessment [48].

It is believed that the National Institutes of Health Stroke Scale (NIHSS) for assessing the severity of neurological impairment is not designed to test global cognitive function. The cognition component of NIHSS (Cog-4) includes assessment of orientation, executive function, language, and attention. However, it yields higher scores for aphasia than for neglect and higher scores related to stroke in the left than in the right hemisphere. Data on using the Cog-4 to assess cognitive status after stroke are inconsistent [49]. The addition of two simple tests of neglect (line cancellation and visual extinction) has been reported to improve the Cog-4 cognitive assessment significantly, and an increase in the difficulty of executive task improves its diagnostic accuracy (AUC 0.81), bringing it closer to that of the MMSE scale (AUC 0.84) [50]. Therefore, without specific scales to assess PSCI, the Cog-4 subscale may reasonably assess cognitive function. In addition, the Cog-4 may even be used as an accurate predictor of dementia (AUC 0.78) when applied in the chronic phase of stroke (18 months after stroke) [50].

In contrast, there is evidence that many stroke survivors with MoCA-defined cognitive deficits could not be diagnosed with Cog-4 due to insufficient test validity and accuracy associated with low sensitivity (36%) despite the favorable specificity (96%) [34] (Table 1). A similar conclusion was drawn by Ankolekar *et al*. [51], who also provided evidence that at day 90 after stroke, the Cog-4 scale cannot be considered a useful cognitive tool as it is highly dependent on stroke location, relates to functional outcome (as a subset of the NIHSS) and has a severe 'floor effect.' These authors recommend using specific and validated assessment tools instead of Cog-4 for establishing post-stroke cognitive status.

Some studies used domain-specific screening tools to achieve better diagnostic and prognostic value of cognitive assessment [52,53] because common cognitive impairments after stroke, e.g., aphasia, neglect, apraxia, visual disturbances, etc., can be evaluated only by tests specially developed for stroke patients. In addition, the impairments in different cognitive domains may have different recovery trajectories and require the implementation of specific rehabilitation procedures.

The Cognitive Assessment Scale for Stroke Patients (CASP) and the Oxford Cognitive Screen (OCS) are specific stroke screening tools. The CASP scale has a unidimensional structure and assesses global cognitive impairment. It has good psychometric properties for cognitive screening in the sub-acute phase of stroke in patients with severe motor aphasia or left hemisphere neglect. Its main disadvantage is that it cannot be applied to patients with visual impairment or severe oral comprehension [54].

On the other hand, OCS rapidly evaluates several cognitive domains (language, memory, attention, calculation, and praxis). An important advantage of the scale is its high sensitivity for stroke-related deficits like aphasia and neglect. The scale has been reported to be reliable and validated as a brief neuropsychological battery [55,56]. Recently, some studies have confirmed its good prognostic value for cognitive and functional outcomes in the chronic phase after stroke [53,57]. However, according to the European Stroke Organisation and the European Academy of Neurology joint guidelines on post-stroke cognitive impairment, there is still scarce data published regarding the OCS diagnostic accuracy when evaluated against reference standards based on clinical diagnosis and/or comprehensive neurophysiological battery [58].

Given the above, no consensus has been reached on which cognitive screening tools are more appropriate for assessing PSCI in specific settings. Although the domain-specific PSCI is a predictor of disability and cognitive dysfunction, nowadays, it remains underdiagnosed. The dynamics of domain-specific impairment have been understudied, as are the rehabilitation outcomes for stroke survivors [59,60]. However, the effect of cognitive impairments on the functional outcome of stroke patients varies depending on the domain affected.

### Validation of cognitive assessment tools

When choosing a test instrument, one should be sure it is reliable and validated [17]. Unfortunately, few studies have been published in which the classic model is used to assess an instrument's accuracy, i.e., an index test against a reference (gold) standard based on an extensive neuropsychological test battery [22]. Using the clinical diagnosis of PSCI/dementia as a reference standard has not always proved appropriate. Moreover, for reliable and valid assessment of the instrument sensitivity and specificity, the index test and the gold standard assessments must be conducted within a short time interval [42]. Indicators such as sensitivity, specificity, positive predictive value (PPV), and negative predictive value (NPV) must ensure that cognitively impaired patients are not omitted. It is recommended that the diagnostic accuracy of the instruments used should reach a sensitivity of ≥80% and a specificity of ≥60%, evaluated in terms of a long-term PSCI diagnosis based on a comprehensive neuropsychological test battery [21,42]. Test sensitivity rather than specificity is the leading factor in initial screening. Although it is essential to report PPV and NPV data, these values may vary depending on the impairment prevalence rate in the population. Therefore, for these parameters to be compared between different studies, they should be calculated based on the sensitivity, specificity, and prevalence of impairment in the study population [42].

## COGNITION ASSESSMENT PROCEDURES WITH COGNITIVE INSTRUMENTS

### Dichotomizing test scores

Apart from using different cognitive tools (screening tests or batteries) in research and clinical practice, the lack of standard approaches to generalizing the data obtained could be problematic.

Results from each cognitive test need to be dichotomized (positive/negative test outcome) based on adjusted norms for age, education, and other factors. Dichotomization is implemented by cut-off values, which vary across studies. Most cut-off values used as criteria for cognitive impairment in more than one cognitive domain are determined based on means and standard deviations (SD) of component/domain test scores ranging from 1.5 SD to 1.98 SD below the age- and education-adjusted control means. The commonly used cut-off values of 1.5 SD and 1.64 SD overestimate the false-positive rate. Also, cognitive impairment was reported with scores below 1 SD or impairment in only one domain, which caused high false positive rates (>20%) [12,17]. However, the parameters like means and standard deviations are defined for normally distributed cognitive data. Studies rarely account for the deviation of cognitive data from a normal distribution. At the same time, demographic factors and the non-parametric distribution of most cognitive data strongly influence the correct determination of cut-off points [61]. Therefore, the cut-off points should be determined based on percentiles [61]. Thus, for instance, the cut-off score of the 5^th^ percentile was found to be the most appropriate in the study of Barbay *et al*. [12], where a 5^th^ percentile threshold applied to the above-mentioned shortened summary cognitive score provided the highest sensitivity and specificity (adjusted true positive rate 43.5% and false positive rate ≤5%, *P* = 0.0001).

Regarding short screening tests, adequate sensitivity and specificity have been found using ROC analysis at different cut-off points, making clinical practice recommendations difficult [33,42,62]. For example, in the study of Salvadori *et al*. [19], a MoCA cut-off value of 21/30 points in the acute phase predicted mid-term PSCI with good sensitivity and specificity (Table 3). A similar MoCA cut-off score (21-22/30) in the acute phase, concerning the mid-term prediction of moderate to severe cognitive impairment, was reported in another study with high sensitivity and NPV [32] (Table 3). MoCA cut-off scores of 23-24/30 in the subacute phase diagnosed cognitive impairment with good sensitivity, NPV, and PPV [31] (Table 3). MoCA cut-off points of 25/30 in the chronic phase of stroke showed good sensitivity and specificity for MCI [36] (Table 3). Many centers recommend a lower MoCA threshold when the scale is administered in stroke assessment [15,22]. A meta-analysis based on four studies showed that a MoCA cut-off of 26/30 had high sensitivity (95%) but low specificity (45%), while lowering the MoCA cut-off (<22/30) slightly reduced sensitivity (84%) but significantly improved specificity (78%) [22] (Table 1). The systematic review of Carson *et al*. [63] recommended a MoCA cut-off value of 23/30, given its overall better diagnostic accuracy (86% correctly classified individuals compared with 78% at a cut-off value of 26 and a lower false positive rate, as defined by Youden index = 0.71). Other investigators [64] chose a MoCA cut-off point <23/30 as a PSCI indicator, citing this review.

**Table 3:** MoCA sensitivity and specificity at different cut-off points

Source	Administration time	Diagnosis	Stroke severity	Cut-offs	Sensitivity, %	Specificity, %	AUC	PPV	NPV	*n*	Reference standard
Salvadori *et al*. (2013) [19]	Acute phase 5-9 days after stroke	Mid-term PSCI impairment 6-9 mos after stroke	Mild to moderate	21/30	91	76	0.90	0.80	0.893	80	Dementia diagnosis
Dong *et al*. (2012) [32]	Acute phase within 14 days after TIA/ICH	Moderate/severe PSCI 3-6 mos after stroke	Mild	21-22/30	88	64	0.85	0.45	0.94	239	Formal neuropsychological battery
Cumming *et al*. (2013) [31]	Subacute phase 3 mo after stroke	PSCI 3 mos after stroke	Mild to moderate	23-24/30	92	67	0.87	0.84	0.82	60	Comprehensive neuropsychological battery
Pendlebury *et al*. (2012) [36]	Chronic phase >1 year after TIA/stroke	MCI >1 year	Mild to moderate	26/30	87	63	-	0.64	0.87	91	NINDS-Canadian Stroke Network Harmonization Standards Neuro
				25/30	77	83	-	0.77	0.83		psychological Battery
				24/30	59	85	-	0.74	0.73		
				23/30	49	90	-	0.79	0.70		
Godefroy *et al*. (2011) [33]	Acute phase <3 weeks after cerebral infarct/hemorrhage	Acute phase PSCI	Mild to moderate	22/30	78	90	0.89	0.94	0.67	95	Comprehensive neuropsychological battery.

ICH, intracerebral hemorrhage; MCI, mild cognitive impairment; n, number of patients; NPV, negative predictive value; PPV, positive predictive value; PSCI, post-stroke cognitive impairment; TIA, transient ischemic attack

Further optimization of the cut-off point for PSCI may be necessary if a study aims to assess a multidomain impairment. A MoCA threshold of <26/30 should be used to detect single-domain/mild cognitive impairment, and an adapted cut-off value (<22/30) could improve the test accuracy in post-stroke multidomain impairments [22].

It should be noted that the screening tests are often assessed separately in research. Few comparative analyses between different tests applied to the same patient samples have been published, possibly because of differences in test designs [58]. Furthermore, performing a summary analysis of the diagnostic accuracy (based on sensitivity and specificity) of tests using meta-analyses is important. However, diagnostic accuracy may vary depending on the cut-off values chosen. Also, education, age, and cultural factors are not always considered when applying standardized cut-offs at the patient level.

### Generalization/integration of test scores by cognitive components/domains

When using a neuropsychological battery, the dichotomized scores of the individual tests should be summarized (integrated) to form the clinical diagnosis (intact cognition or cognitive impairment).

Different types of generalizations have been proposed in the literature, such as the number of negative test scores, the number of impaired domains, the mean score of different cognitive domains (e.g., language, visuospatial abilities, memory, executive functions), and the global summary score (e.g., mean of all cognitive scores after converting the raw scores into a standard metric, such as a z-score). Sometimes, a single negative test score was considered sufficient to classify a patient with cognitive impairment. Other procedures focus on the cognitive domains (assessed with one or more tests) and classify the presence of cognitive impairment in cases of one or more impaired domains. In clinical practice, the judgment about cognitive impairment is usually based on the number of tests with negative scores.

However, it should be noted that PSCI criteria, based on multidomain cognitive assessment, improve sensitivity but may result in a high false positive rate, i.e., a high proportion of individuals with intact cognition but negative test scores [19,21,61]. It is, therefore, necessary to find the most favorable balance between specificity and sensitivity as a function of the number of tests. Even when the false positive rate for a particular procedure is less than 5%, using different methods to analyze and summarize cognitive data may influence sensitivity significantly, and the difference can be as big as threefold between one procedure and another [61].

In conclusion, adopting a standardized approach for test scoring, dichotomizing, and integrating individual test scores across test batteries may improve the accuracy of diagnosis, prognosis, and prevalence assessment of cognitive impairment [61,65,66].

### Potential for standardization/harmonization of cognitive status assessment

Several studies have addressed the issues of developing optimized criteria for assessing post-stroke cognitive status [7,33,42]. It is believed that in routine practice, the cognitive screening tool used should readily identify patients at risk of cognitive impairment. When using neuropsychological batteries, it is important to assess fixed cognitive domains (e.g., five-speech, visual-constructive abilities, memory, speed of action, and executive abilities) in addition to depression and behavioral changes [7]. The procedure for processing the combination of cognitive scores obtained with these batteries is also recommended to be harmonized. When the outcome measure (impairment/intact cognition) from administering a neuropsychological battery of tests is based on the number of 'negative' scores on individual tests, as in clinical practice, this number should be adjusted for the number of tests. However, such an adjustment reduces sensitivity and the ability to detect selective impairments. Published evidence has shown that determining a global summary score (obtained, for example, from a mean z-score) allows one to distinguish patients from controls even when the impairment affects only one cognitive process [61]. Furthermore, the highest sensitivity has been achieved using this global summary score. However, the authors recommend that this assessment be based on stroke-specific tests.

The literature also recommends adopting standard criteria for mild and severe post-stroke cognitive impairment, such as those proposed by the VASCOG group [60]. According to the VASCOG criteria, harmonizing the threshold to define cognitive impairment is essential, given that it significantly affects the diagnosis and prediction of post-stroke cognitive deficits [7]. In particular, the cut-off scores should be adjusted for age, education, premorbid intelligence, stroke characteristics, etc., since normative studies of rating scales clearly show the broad impact of demographic and cultural factors on their performance [21, 42,67]. Therefore, the formation of standardized and sufficiently large normative samples has been proposed, stratified by age and education in the countries where these procedures will be implemented. In addition, Godefroy *et al*. [61] provided a rationale for calculating the size of normative populations required to ensure a 95%CI of the 5^th^ percentile below a given value, thus establishing an approach to harmonize the diagnosis of neurocognitive disorders and reduce the heterogeneity between studies in terms of reported PSCI prevalence and prognosis.

An essential point in determining the accuracy and generalizability of the results obtained is implementing an approach to account for the missing data of patients who could not complete the index test and the reference standard because of communication problems or confusion [68]. Excluding these data from the analysis limits external validation [12].

Another source of heterogeneity is the time point after stroke for the test assessment. Authors recommend investigating the potential effect of time after stroke on the sensitivity and specificity of assessment instruments [42]. In this regard, there is published evidence in favor of acute phase-cognitive assessment [22]. Future research is also needed to confirm which assessment tools are appropriate for initial screening and long-term assessment of post-stroke cognitive status. Over the years, a number of cognitive tools have been developed and studied. Diagnostic criteria for defining PSCI have been proposed, and some guidelines for assessing cognitive status in stroke patients have been designed. However, systematic reviews and meta-analyses have encountered certain difficulties in generalizing the results from different studies, thus preventing the formulation of recommendations for choosing the best cognitive assessment tool/procedure in the context of a specific situation.

Harmonizing post-stroke assessment is a long-standing and challenging problem, still waiting to be resolved. To select the most appropriate tool and procedure in a given clinical context, clinicians can use evidence-based guidelines to assess cognitive impairments after stroke. In particular, analyzing the diagnostic accuracy of cognitive instruments with a focus on metrics such as sensitivity and specificity may be useful. The potential consequences of false positive and false negative diagnoses should also be considered.

Furthermore, the accuracy of screening instruments has often been assessed in isolation. Therefore, comparative analyses of the diagnostic accuracy of different screening tools used in a large cohort under specified conditions - study design, stroke phase, and cut-off values, are needed. The development of cognitive tools, validated in independent multicenter cohorts, that can assess the risk of PSCI is also crucial.

Systematic reviews and meta-analyses highlight too many small studies with methodological limitations and a high risk of bias as serious drawbacks. In this regard, reducing patient dropout rates, correctly applying statistical analyses by considering the type of cognitive data distribution, using statistical methods accounting for missing data, blind interpretation of the index test or reference standard, etc., are essential. It should also be kept in mind that stroke-related deficits, such as neglect and aphasia, as well as demographic factors, such as education, language, or culture, can render the results obtained from cognitive screening instruments misleading. On the other hand, evidence-based clinical practice can improve guidelines for clinical assessment of PSCI.

Given the existing clinical stroke guideline recommendations for acute-phase cognitive assessment, an optimal approach for its implementation in clinical settings should be sought. A contradiction exists between the need to use a detailed and clinically sensitive cognitive tool and the requirements for the feasibility of cognitive assessments in acute clinical settings. Also, cognitive tools should be freely available and applicable in routine practice.

## PROGNOSTIC VALUE OF COGNITIVE ASSESSMENT

Some studies have highlighted the clinical efficacy of early cognitive testing (in the acute and subacute phase) for mid- and long-term PSCI prognosis. For this purpose, as noted above, brief cognitive tests are appropriate for initial screening in the acute phase.

**Figure 3:**
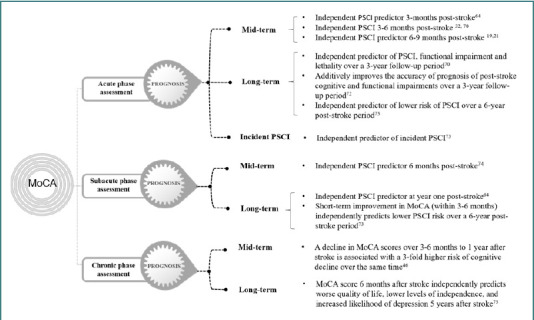
Prognostic value of Montreal Cognitive Assessment. MoCA, Montreal Cognitive Assessment; PSCI, post-stroke cognitive impairment

Encouraging data about the good prognostic accuracy and validity of the MoCA scale in acute stroke patients have been published in the last decade [62,69]. Accumulating evidence suggests that acute phase cognitive assessment with MoCA predicts mid- and long-term cognitive and functional status and survival after stroke (Figure 3, Table 4). For example, the baseline MoCA score is independently associated with PSCI 3-6 months post-stroke [32,70]. MoCA in hospitalized patients with mild stroke predicts 3-month PSCI (OR = 0.67) [71] (Table 4). In the study of Salvadori *et al*. [19], MoCA scoring in the acute phase of stroke was reported as a good predictor of mid-term (6-9 months post-stroke) PSCI, regardless of age, education, functional and cognitive premorbid status, stroke severity, and history of lacunar infarcts (OR = 1.4) (Table 4). Moreover, according to the authors, if MoCA is inapplicable in the acute phase of stroke to assess cognition, this indicates further cognitive deterioration [19]. Zietemann *et al*. [72] also found that the baseline MoCA scores of patients without dementia before stroke, regardless of age, premorbid cognitive status, and NIHSS at admission, predicted cognitive impairment as defined by a battery of neuropsychological tests (OR = 5.30) and Clinical Dementia Rating ≥0.5 (OR = 2.53) over a 3-year follow-up period (Table 4). MoCA scores also predicted functional impairment as defined by Modified Rankin Scale (mRS)>2 (OR = 5.03) and Instrumental Activities of Daily Living (IADL)<8 (OR = 2.48), as well as lethality (HR = 7.24) over the same period [72]. An additive predictive value of the MoCA scale was found using ROC analysis. MoCA increased the area under the ROC curve for predicting cognitive dysfunction (AUC 0.81 versus AUC 0.72 on neuropsychological testing) and functional impairment (0.88 versus 0.84 on mRS score >2) (Table 4). In the long term, Zhao *et al*. [73] found that baseline MoCA scores (OR = 0.66) were an independent predictor of lower risk of PSCI over a 6-year post-stroke period.

**Table 4:** Prognostic value of cognitive assessment

Source		*n*	Stroke type	Cognitive tool	Time to administer	Statistical method	Prognostic value of cognitive assessment				Conclusion
							Predictor	Outcome variable(s)			
Jacquin A *et al*. (2014) [71]		220	IS, hemorrhagic	MMSE MoCA	5 d,3 mos	Multivariable logistic regression	MMSE <26, 5 d	3 mo-PSCI, OR(95%CI)(adj)=O.63(0.54-0.74), P<0.0001		MMSE and MOCA during the acute phase of stroke are	
							MoCA <26,5 d	3 mo-PSCI, OR(95%CI)(adj)=O.67(0.59-0.76), P<0.0001		independently associated with PSCI 3 mos after stroke.	
Jokinen H *et al*. (2015) [9]		409	IS	NAB	3 mos (mean)	Logistic regression	Memory functions Visuoconstructional/spatial Executive functions/attention Aphasia Reading and writing Abstract reasoning	15 mo-functional dependence (mRS>2) OR(95% Cl)=2.2(1.2-3.9), P=0.008 OR(95% Cl)=5.1(2.7-9.1), P<0.001 OR(95% CI)=3.2(1.8-5.7), P<0.001 OR(95% Cl)=2.1 (1.1—3.9), P=0.017 OR(95% Cl)=2.3(1.2-4.3), P=0.011 OR(95% Cl)=2.3(1.3—4.2), P=0.006)		Complex cognitive abilities are compromised 3 mos post-stroke. PSCI is significantly associated with poor functional outcomes during long-term follow-up.	
Milosevich ET *et al*. (2024) [53]		430	IS, hemorrhagic and mixed	OCS	<2 wks, 6 mos	Hierarchical multivariable regression	0CSS2 weeks Domain-specific PSCIS2 wks in memory in language in praxis	6 mo-PSCI, R^2^(adj)=0.298, P<0.0001 6 mo-PSCI, R^2^(adj)=0.298, P<0.0001 ß(SE)=0.116(0.027), P<0.0001 ß(SE)=O.O95(O.O27), P<0.0001 ß(SE)=0.084(0.028), P<0.002		Acute PSCI is strongly associated with both PSCI severity at 6 mos and domain-specific PSCI, explaining -30% more outcome variance than demographic and clinical factors.	
Narasimhalu K. *et al*. (2011) [5]		419	IS, TIA	NAB, CIND >1 domain CIND-mild (1-2 domains) CIND-moderate (3-6 domains)	3 mos, 3.2 yrs mean follow-up	Cox regression	CIND CIND-mild CIND-moderate CIND-moderate Baseline visuomotor speed	**For a mean of 3.2 years after stroke:** Dependency (m RS, good outcome (0 -2) and bad outcome (3- 6) HR (adj) (95% CI)=3.77(1.52-9.37) HR(adj) (95% CI)=4.05(1.58-10.4) HR(adj) (95% Cl)=3.41 (1.27-9.13) Death, HR(adj) (95% Cl)=3.81 (1.14-12.8) Dependency, HR(adj) =3.49, P<0.002		CIND predicts dependency and death, while CIND severity - poor survival.	
Rohde D *et al*. (2019) [75]		226	IS	MoCA	6 mos	Multivariable logistic and linear regressions	MoCA, 6 mos	**5 years after stroke:** IADL independence, B(95% Cl) (adj)=-3.6O5 (5.705-1.505), p<0.01 Quality of life (SSQOL), B(95% Cl) (adj)=-0.595 (0.943-0.248), p<0.01 Depression (SESDS), OR(95% Cl) (adj)= 4.60 (1.22 -17.40), p<0.05		PSCI post-stroke is associated with worse outcomes.	
Salvadori E *et al*. (2013) [19]	80		is, hemorrhagic	MoCA	5-9d, 6 - 9 mos	Multivariate logistic regression ROC analysis	Baseline MoCA Cut-off of 21		PSCI, OR(95 % Cl)(adj)=1.4(1.1-1.8) PSCI, AUC=0.902, P<0.001, sensitivity 91 %, specificity 76%, PPV 0.80, NPV 0.89	Baseline MoCA is a good mid-term independent PSCI predictor The cut-off of 21 showed good specificity and PPV	
Tan HH *et al*. (2017) [46]	400		IS, TIA	MoCA decline by 2 pts over two consecutive time points	< wks, 3-6 mos, 1 yr	Logistic regressions	Change in MoCA from baseline to 3-6 mos		Decline in MoCA scores (3-6 mos to 1 year) OR (95% Cl)=3.21 (1.45-7.08), P<0.01).	The decline in MoCA scores from 3-6 mos to 1 yr has a 3-fold higher risk for decline in the diagnosis transitional status	
Zhao X *et al*. (2021) [73]	244		IS, TIA	MoCA NAB, 1.5 SD	< 14 d, 3-6 mos, 1 yr, 3/4 yr, 5 and 6 yrs	GEE model Kaplan-Meier survival analysis Multiple logistic regressions	Baseline MoCA MoCA improvement (3-6mos) MoCA improvement <1 yr Baseline MoCA		**Over a 6-year follow-up period:** PSCI, OR(95 % CI)(adj)=0.66(0.59-0.74]), PSCI, OR(95%CI)(adj)=0.80(0.71—0.89]) PSC1 OR(95%C 1) (a d j)=0.86 (0.76-0.96) Incident PSCI, OR(95%CI)(adj)=0.76(0.61-0.96)	Baseline and short-term MoCA improvement are independent predictors of long-term PSCI. MoCA improvement (<1 yr) is associated with longitudinal cognitive improvement.	
Zhu Y *et al*. (2020) [70]	229		IS	MoCA MMSE NAB	<2 ws <2 ws 3-6 mos	ROC analysis	Baseline MoCA <21 Baseline MMSES27		PSCI (3-6 mos) Sensitivity 64 %, specificity 90%, PPV 0.91, NPV 0.59, accuracy 0.73 Sensitivity 68 %, specificity 82%, PPV 0.87, NPV 0.60, accuracy 0.73	Within 2 wks of stroke, MMSE and MoCA have similar predictive values for PSCI 3-6 mos after stroke.	
Zietemann V *et al*. (2018) [72]	274		IS, hemorrhagic	MoCA <26 NAB <1.5 SD, >1 domain CDR score >0.5	<1 wk, 6,12 and 36 mos	Linear, logistic, and Cox regressions, ROC analysis	Baseline MoCA <26		**Over a 36-mo follow-up period:** PSCI (NAB), OR(95%C 1)(adj)=5.3O(2.75-10.22) PSCI (CDR), OR(95%CI)(adj)=2.53(1.53-4.18) Functional impairment (mRS >2) OR(95%CI) (adj)=5.O3(2.2O-11.51) Functional impairment (IADL<8), OR(95%CI) (adj)=2.48 (1.40-4.38) Increased mortality, HR(95%Cl)(adj)=7.24(1.99- 26.35) MoCA increased AUC to predict PSCI (NPS 0.81 vs 0.72, p=0.01) and functional impairment (0.88 vs 0.84, p=0.047).	MoCA is a good long-term independent predictor of cognitive and functional outcomes and mortality after stroke.	

Adj, adjusted; AUC, area under the curve; CDR, Clinical Dementia Rating scale; Cl, confidence interval; CIND, cognitive impairment no dementia; d(s), day(s); GEE, generalized estimating equation; HR, hazard ratio; IADL, Instrumental Activity of Daily Living; IS, ischemic stroke; NPV, negative predictive value; MMSE, Mini-Mental State Examination; MoCA, Montreal Cognitive Assessment; mo(s), month(s); mRS, modified Rankin scale; n, number of patients; NAB, Neuropsychological Assessment Battery; NEADL, Nottingham Extended Activities of Daily Living Scale; DCS, Oxford Cognitive Screen; OR, odds ratio; PPV, positive aredictive value; PSCI, post-stroke cognitive impairment; SESDS, Center for Epidemiologic Studies Depression Scale; ROC, Receiver Operating Characteristic; SD, standard deviation; SSQOL, Stroke Specific Quality of Life Scale; TIA, transient ischemic attack, w(s), week(s).

There was also evidence that baseline MoCA score was independently associated with incident PSCI (OR = 0.76) after adjustment for demographic factors, education, vascular risk factors, premorbid cognitive status, and NIHSS stroke severity scale [73] (Table 4).

In the subacute phase of stroke, MoCA scores also predicted mid- and long-term PSCI among stroke patients (Figure 3). In particular, MoCA in the subacute phase of ischemic or hemorrhagic stroke (2 months after stroke) was an independent predictor (β = 0.725; *P* < 0.001) of cognitive impairment in the sixth month after stroke [74]. The MoCA global cognition score in the subacute phase (3 months after stroke) is one of the most significant and independent predictors of PSCI at year one after stroke [63]. Furthermore, Zhao *et al*. [73] found that the short-term improvement in MoCA (within 3-6 months) (OR = 0.80) was an independent predictor of lower risk of long-term PSCI over a 6-year post-stroke period (Figure 3, Table 4). The authors also concluded that an increase in MoCA score within one year was associated with long-term improvement in cognitive function (OR = 0.86).

Such an increase in MoCA scale scores is associated with brain plasticity, and cognitive improvement over a short interval (within one year) could be an early indicator of long-term cognitive stability [73]. On the other hand, a decline in MoCA scores over 3-6 months to 1 year after stroke was associated with a 3-fold higher risk (OR = 3.21) of cognitive decline over the same period [46] (Table 4). Such a reduction can serve as a potentially efficient indicator of the necessity to conduct further neuropsychological testing of stroke patients [46]. In the study of Rohde *et al*. [75], MoCA score during the early chronic phase (6 months after stroke) was an independent predictor of worse quality of life (B = 0.595), lower levels of independence (B = 3.605), and increased likelihood of depression (OR = 4.60) in the long term (5 years after stroke) [75] (Figure 3, Table 4).

The published evidence cited above suggests that cognitive assessment (in the acute, subacute, and chronic phases of stroke) with MoCA can predict long-term cognitive and functional status, supporting the routine use of MoCA in stroke patients. Indeed, the prognostic value of MoCA scores combined with other PSCI determinants for early identification of stroke patients at the highest risk for mid- and long-term cognitive decline needs to be explored on a larger scale.

Concerning the other commonly used rating scale, MMSE (AUC, 95%CI = 0.821, 0.743–0.898), if administered within two weeks of stroke, has a prognostic value similar to that of MoCA (AUC, 95%CI = 0.809, 0.725–0.892) for PSCI in the mid-term (3-6 months after mild stroke onset) (*P* = 0.75) [70]. MMSE scores during hospitalization for mild stroke have been associated with 3-month PSCI (OR = 0.63) [71] (Table 4). Published evidence shows that the MMSE memory subscale has predictive value for cognitive status one year after stroke [26]. In the long term, MMSE (cut-off value 23–24/30) is suitable for predicting dementia 24 months after stroke (AUC 0.94, sensitivity 96%, specificity 83%) but could not be used to predict cognitive deterioration or improvement over time [30]. Moreover, published results suggest that the MMSE is not a significant predictor of cognitive status or has insufficient predictive validity [62].

Another screening tool, the IST scale (≤28), predicts cognitive status one year poststroke [26]. Also, the 4-item NIHSS subset, Cog-4, could be used as an accurate predictor of dementia 18 months after stroke (AUC 0.78 vs. diagnosis of severe cognitive impairment) [50].

Regarding domain-specific cognitive impairment, visuomotor speed was reported as an independent predictor of functional disability after stroke (HR = 3.49) [5] (Table 4). In another study, all domain-specific cognitive impairments assessed with the neuropsychological test battery of the Helsinki Stroke Aging Memory Study and analyzed one by one, except for dyscalculia, were significantly associated with functional dependence (mRS>2) at 15-month follow-up of stroke patients regardless of age, sex, years of education, and NIHSS [9] (Table 4). The assessment of cognitive abilities using the stroke-specific OCS scale in the acute phase of the disease was a strong and independent predictor of long-term functional outcomes as assessed with the Stroke Impact Scale 3.0 and the Geriatric Depression Scale [57]. Furthermore, the predictive ability of the scale is significantly improved when applied in combination with some demographic factors and the NIHSS. A recent study has shown that domain-specific OCS screening predicts cognitive outcomes in the early chronic phase [53] (Table 4). Impairments, particularly in memory, language, and praxis, predict the severity of cognitive impairment six months after stroke (Table 4). Studies using the OCS are considered less biased because of the smaller number of excluded stroke patients, generally assumed to be untestable. However, no consensus has been reached on which scale, MoCA or OCS, is more sensitive [52, 56] or informative [76] for recording PSCI in acute stroke settings. Further validation of both tests with larger sample sizes is needed.

The data above suggests that cognitive diagnosis of stroke patients may help identify individuals at high risk of developing PSCI, functional dependence, and poor survival.

It should be noted, however, that cognition may vary between the subacute and chronic stages of stroke, given the evidence of delayed-onset PSCI and the potential for cognitive improvement over time due to improved cerebral perfusion [77]. Therefore, cognitive assessment in the acute phase of a stroke may sometimes be an insufficiently reliable predictor of long-term cognitive status [2].

## CONCLUSION

Adopting standard criteria for diagnosing mild and severe post-stroke cognitive impairment would be helpful in routine clinical practice. Published studies highlight the clinical benefit of early cognitive assessment for the mid- and long-term PSCI prognosis. Regarding the diagnostic accuracy of the instruments used, a sensitivity of ≥ 80% and a specificity of ≥ 60% in terms of a long-term PSCI diagnosis based on a comprehensive neuropsychological test battery are recommended. PPV and NPV should be calculated by considering sensitivity, specificity, and the prevalence of impairments in the study population. A threshold score of the fifth percentile below the age- and education-adjusted control mean is considered most appropriate. Evidence-based, validated, reliable, and harmonized post-stroke cognitive assessment procedures could improve the ability to objectively analyze and summarize results published in the scientific literature regarding PSCI diagnosis, prevalence, and prognosis.
